# Modeled Cost-Effectiveness of a Rideshare Program to Facilitate Colonoscopy Completion

**DOI:** 10.1001/jamanetworkopen.2025.30515

**Published:** 2025-09-04

**Authors:** Rachel B. Issaka, Laura Matrajt, Pedro Nascimento de Lima, Carolyn M. Rutter

**Affiliations:** 1 Public Health Sciences Division, Fred Hutchinson Cancer Center, Seattle, Washington; 2 Hutchinson Institute for Cancer Outcomes Research, Fred Hutchinson Cancer Center, Seattle, Washington; 3 Division of Gastroenterology, University of Washington School of Medicine, Seattle; 4 Vaccine and Infectious Disease Division, Fred Hutchinson Cancer Center, Seattle, Washington; 5 Department of Applied Mathematics, University of Washington, Seattle; 6 Engineering and Applied Sciences Department, RAND Corporation, Arlington, Virginia

## Abstract

**Importance:**

In colorectal cancer (CRC) screening, too many patients fail to receive follow-up colonoscopy after an abnormal fecal immunochemical test (FIT), and transportation is a frequently reported barrier.

**Objective:**

To determine the outcomes and cost-effectiveness of providing a rideshare intervention to patients with abnormal FIT results.

**Design, Setting, and Participants:**

The CRC-Simulated Population Model for Incidence and Natural History microsimulation model was used to simulate the outcomes and cost-effectiveness of a rideshare intervention to improve colonoscopy completion in a population-based CRC screening program. Cohorts were adherent to annual FIT-based screening; baseline analyses assumed that 35% would complete a follow-up colonoscopy. Data were analyzed from November 14, 2023, to July 8, 2025.

**Intervention:**

A $40 or $100 rideshare to increase completion of follow-up colonoscopy.

**Main Outcomes and Measures:**

Lifetime outcomes included the number of CRC cases, deaths, and life-years gained (LYG) per 1000 people screened and costs associated with improved completion of a colonoscopy after an abnormal FIT result.

**Results:**

Four single-age cohorts (ages 45, 55, 65, and 70 years on January 1, 2024) of 10 million people each were simulated. In cohorts with similar sex distribution as the US population (aged 45 years, 50.0% male; aged 55 years, 49.4% male); aged 65 years, 48.0% male; and aged 70 years, 46.9% male), compared with no intervention, using a rideshare intervention starting at age 45 years that costs $100 per ride to increase colonoscopy completion from 35% to 70% was associated with a reduction in CRC cases per 1000 by 26.3% (30.7 vs 41.6 cases per 1000), CRC deaths per 1000 by 32.5% (9.8 vs 14.6 cases per 1000), 24.9 LYG per 1000, and at $100 per ride cost $43 308 per 1000 people screened and saved $330 587 per 1000 people screened.

**Conclusions and Relevance:**

In a microsimulation model, increasing colonoscopy completion in a population with abnormal FIT results via a rideshare intervention was cost saving up to $100 per ride due to the combined outcome of cancer prevention and early detection.

## Key Points

**Question:**

What is the modeled cost-effectiveness of providing rideshare for colonoscopy to patients with abnormal fecal immunochemical test (FIT) results?

**Findings:**

In this decision-analytic model study with 4 simulated cohorts of 10 million people, using a $100 rideshare intervention starting at age 45 years to increase colonoscopy completion from 35% to 70% cost $43 308 per 1000 people screened and was associated with saving $330 587 per 1000 people screened and 24.9 life-years gained per 1000 people screened compared with no intervention.

**Meaning:**

These findings suggest that increasing colonoscopy completion in a population with abnormal FIT results via a rideshare intervention is cost saving at $100 per ride due to detecting earlier-stage colorectal cancer and preventing colorectal cancer through detection of precancerous lesions.

## Teaser

This decision analytical model study evaluates the cost-effectiveness of providing a rideshare intervention to help patients with abnormal fecal immunochemical test results attend follow-up screenings.

## Introduction

Colorectal cancer (CRC) is the second highest cause of cancer deaths in the US and is projected to be the leading cause of cancer deaths in adults under age 50 years by 2030.^1^ CRC is largely preventable through screening, yet 58% of the US population aged 45 to 75 years and only 41% of adults in federally qualified health centers (FQHCs) are up to date with CRC screening; well below the National Colorectal Cancer Roundtable screening goal of 80%.^2^ To improve screening, especially in FQHCs and other resource-constrained settings (collectively referred to as safety-net health care settings), stool-based tests, including the fecal immunochemical test (FIT) are commonly used. Stool-based tests are a 2-step screening strategy; therefore, abnormal results must be followed by a colonoscopy to realize the benefit of screening. While 1 in 6 adults use stool-based tests for CRC screening, follow-up colonoscopy rates are suboptimal and range from 18% to 75% within 1 year of the abnormal result, with higher rates of follow-up observed in insured populations.^3,4^ Failure to complete a follow-up colonoscopy carries a 3-fold increased risk for advanced-stage CRC and a 2-fold increased risk of dying from CRC.^5^

In the US, most colonoscopies are completed with procedural sedation. Due to concerns about delayed psychomotor function, endoscopy units often require that patients have a chaperone drive them home after receipt of sedation.^6^ This is a common barrier to follow-up colonoscopy completion, especially in safety-net health care settings due to higher social needs. We reported that 25% of individuals with abnormal FIT results delayed or missed a follow-up colonoscopy due to lack of transportation and/or chaperone.^7^ A meta-analysis of 7 studies^8^ found that providing nonemergency medical transportation, including rideshare for multiple medical indications, reduced missed appointments by 37%. While many barriers to follow-up colonoscopy completion exist, addressing transportation barriers should be a priority given its potential to improve CRC outcomes.

We previously reported that a rideshare intervention after procedural sedation was safe, acceptable, and feasible in a safety-net health care system.^9^ In qualitative analysis, we also found that patients with transportation barriers who would have otherwise not completed a follow-up colonoscopy did so when offered the rideshare intervention. Despite legal and risk concerns for use of rideshare in settings that administer sedation, to date there is a paucity of legal literature on the topic. This coupled with an increased demand for colonoscopy to prevent and surveil CRC has led to growing interest in how rideshare could be harnessed to address transportation barriers to care.^6^ Therefore, the objective of this study was to estimate the outcomes and cost-effectiveness of providing rideshare to populations with abnormal stool-based test results using the CRC Simulated Population Model for Incidence and Natural History (CRC-SPIN) microsimulation model. We compared scenarios with the rideshare intervention compared with no intervention at varying costs. Our findings are designed to inform a potential Centers for Medicare and Medicaid Services coverage decision for similar rideshare interventions.

## Methods

### Setting

This analysis focused on projecting outcomes for patients using the Fred Hutch/University of Washington Medicine Population Health CRC Screening Program. Details of the program are summarized elsewhere.^10^ In brief, patients eligible for CRC screening receive annual mailed FIT outreach and navigation to assist with screening completion and follow-up colonoscopy completion for abnormal results. In 2021, at the program’s inception, 35% of patients with an abnormal stool-based test completed a follow-up colonoscopy within 1 year of this result. The cohort-level decision model did not use patient data and is therefore not considered human participants research that requires institutional review board approval in accordance with 45 CFR §46. This decision-analytic microsimulation study was conducted in accordance with the Consolidated Health Economic Evaluation Reporting Standards (CHEERS) 2022 reporting guidelines.^11^

### Microsimulation Model

We used a validated model that is part of the Cancer Intervention and Surveillance Modeling Network.^12,13^ In brief, present model is an individual-level microsimulation model that describes transitions through the CRC disease process for each person modeled. All individuals in the model start in a disease-free state at age 10 years. As they age, they can experience different disease states: development of precursor lesions (adenomas) and transition to asymptomatic CRC, to clinical CRC, and to CRC or other-cause death. CRC screening interrupts this process (eFigure in Supplement 1). The model has been calibrated to clinical data and has been extensively used to investigate CRC screening schedules and inform recommendations by the US Preventive Services Task Force (eTables 1 and 2 in Supplement 1).^14,15^

### Simulated Cohort

We simulated 4 single-aged cohorts of 10 million cancer-free individuals aged 45, 55, 65, or 70 years who underwent annual FIT screening (starting at age 45 years for the 45-year-old cohort and starting at age 50 years for the other cohorts). The proportion of men in each cohort was set to the age-specific proportion of men in the US population in 2020.^16^ We simulated a lifetime time horizon starting on January 1, 2024, with individuals starting at their cohort age until death, either from CRC or other causes. During this period, individuals were assumed to have yearly FIT tests until age 76 years. Because we are interested in evaluating the outcomes of increased adherence rates to follow-up colonoscopy, our analyses focused on individuals with perfect adherence to FIT screening but imperfect adherence to follow-up of abnormal tests and assumed that people with findings at follow-up colonoscopy were perfectly adherent to adenoma surveillance guidelines. Based on health system data and several studies that have reported follow-up colonoscopy completion rates ranging from 18% to 75% (with higher rates of follow-up observed in insured populations), we simulated a baseline scenario assuming 35% follow-up colonoscopy completion before the rideshare intervention.^3,4^ We then compared this baseline scenario with scenarios simulating rideshare interventions that would increase follow-up colonoscopy completion, with resulting follow-up rates ranging from 40% to 100%.

### Statistical Analysis

We reported clinical outcomes including the number of CRC cases and deaths per 1000 people screened projected over the remaining lifetime of simulated cohorts. We also reported outcomes including life-years gained (LYG) relative to not completing a follow-up colonoscopy over the cohorts’ remaining lifetime and overall costs of CRC care. These costs of care include FIT costs, colonoscopy costs (both follow-up and surveillance colonoscopies), costs derived from colonoscopy complications, treatment costs for different CRC stages, and the costs associated with the rideshare program. We assumed that the rideshare program would be applicable both to follow-up colonoscopies and surveillance colonoscopies, so the costs reported here include both. We assumed a $40 per ride cost reflective of the mean cost per ride from our prior work and a $100 per ride cost—an adjustment to reflect potential costs in more expensive markets or areas with limited rideshare availability. For each age cohort, we also calculated the maximum cost per rideshare to determine the cost at which the intervention was no longer cost-effective. Costs were applied to both screening and surveillance colonoscopies for everyone who received a procedure. All costs and benefits were discounted at 3% per year and were calculated from the payer perspective, as we assume that insurers would bear rideshare costs. Data were analyzed in R version 4.4.0 (R Project for Statistical Computing) from November 14, 2023, to July 8, 2025.

## Results

**Figure 1.**
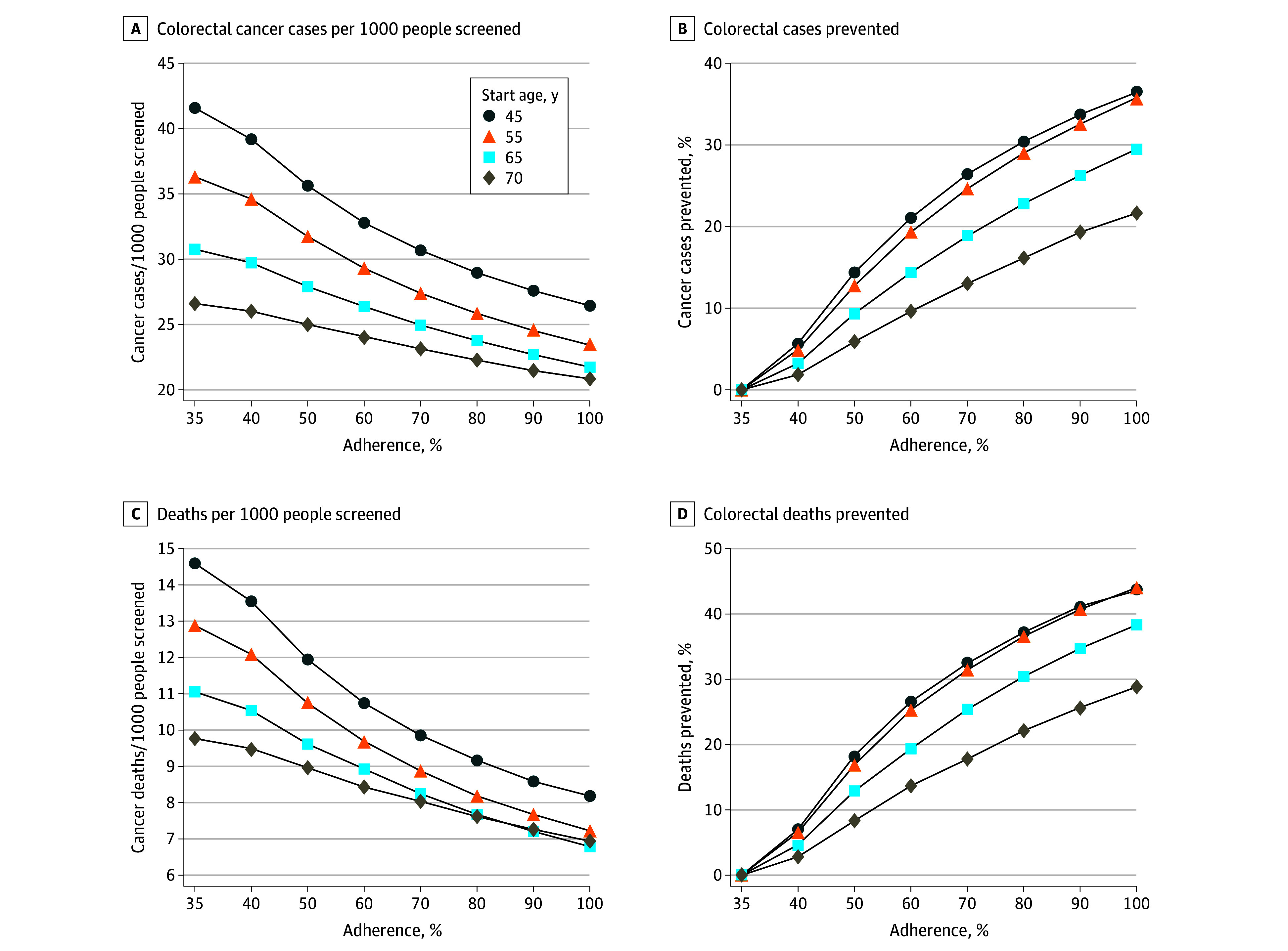
Colorectal Cancer Cases and Deaths Prevented by Varying Adherence to Follow-Up Colonoscopy Completion

The study simulated annual FIT screening in 4 single-aged cohorts of 10 million cancer-free individuals aged 45, 55, 65, or 70 with 50.0%, 49.4% 48.0% and 46.9% of the cohorts being male, respectively. Assuming a screening population of patients aged 45 years, a $40 rideshare intervention that increased colonoscopy completion by 15 percentage points (pp) (35% to 50%) was associated with a reduction in CRC cases per 1000 by 14.4% (35.6 vs 41.6 cases per 1000) and CRC deaths per 1000 by 18.5% (11.9 vs 14.6 cases per 1000), resulting in 13.4 LYG per 1000. An intervention that doubled colonoscopy completion in patients aged 45 years (from 35% to 70%) was associated with a reduction in CRC cases per 1000 by 26.3% (30.7 vs 41.6 cases per 1000), CRC deaths per 1000 by 32.9% (9.8 vs 14.6 cases per 1000) and resulted in 24.9 LYG per 1000 (Figure 1 and Table). Model-estimated values with 95% credible intervals (CrIs) are summarized in eTable 3 in Supplement 1.

**Table.** Colorectal Cancer Cases and Deaths Prevented, Life-Years Gained, Net Costs, and Maximum Cost Per Ride by Varying Colonoscopy Adherence Across Multiple Age Groups

Starting age, y, and outcome	Adherence to colonoscopy, %							
	35	40	50	60	70	80	90	100
45								
Cases per 1000	41.6	39.2	35.6	32.8	30.7	28.9	27.6	26.4
Deaths per 1000	14.6	13.6	11.9	10.7	9.8	9.2	8.6	8.2
Life-years gained per 1000	[Reference]	5.1	13.4	19.8	24.9	28.9	32.3	34.7
Ride cost per 1000 ($40/ride)	[Reference]	3107	8564	13 230	17 323	20 960	24 274	27 294
Ride cost per 1000 ($100/ride)	[Reference]	7767	21 410	33 076	43 308	52 400	60 685	68 235
Maximum cost per ride	[Reference]	156	316	392	418	436	427	424
Net costs per 1000 ($40/ride)	[Reference]	−80 953	−204 640	−297 100	−356 572	−408 054	−431 696	−457 219
Net costs per 1000 ($100/ride)	[Reference]	−76 293	−191 794	−277 254	−330 587	−376 614	−395 285	−416 278
55								
Cases per 1000	36.2	34.5	31.7	29.2	27.4	25.7	24.5	23.4
Deaths per 1000	12.9	12.0	10.7	9.6	8.8	8.2	7.6	7.2
Life-years gained per 1000	[Reference]	3.2	8.5	13.1	16.5	19.3	21.7	23.8
Ride cost per 1000 ($40/ride)	[Reference]	2259	6306	9839	12 960	15 766	18 287	20 620
Ride cost per 1000 ($100/ride)	[Reference]	5647	15 765	24 599	32 400	39 414	45 717	51 551
Maximum cost per ride	[Reference]	145	305	393	429	449	457	453
Net costs per 1000 ($40/ride)	[Reference]	−45 350	−124 954	−193 652	−242 868	−283 519	−315 042	−336 551
Net costs per 1000 ($100/ride)	[Reference]	−41 962	−115 495	−178 893	−223 427	−259 871	−287 612	−305 620
65								
Cases per 1000	30.8	29.7	27.9	26.3	25.0	23.8	22.7	21.7
Deaths per 1000	11.0	10.5	9.6	8.9	8.3	7.7	7.2	6.8
Life-years gained per 1000	[Reference]	1.5	4.0	6.2	8.2	9.9	11.3	12.5
Ride cost per 1000 ($40/ride)	[Reference]	1338	3816	6085	8136	10 010	11 741	13 356
Ride cost per 1000 ($100/ride)	[Reference]	3345	9541	15 212	20 339	25 026	29 352	33 390
Maximum cost per ride	[Reference]	160	312	395	450	474	483	498
Net costs per 1000 ($40/ride)	[Reference]	−22 576	−62 046	−99 750	−135 476	−163 522	−185 845	−210 689
Net costs per 1000 ($100/ride)	[Reference]	−20 570	−56 321	−90 623	−123 272	−148 506	−168 234	−190 654
70								
Cases per 1000	26.6	26.1	25.0	24.0	23.1	22.3	21.5	20.8
Deaths per 1000	9.8	9.5	9.0	8.4	8.0	7.6	7.3	7.0
Life-years gained per 1000	[Reference]	0.7	1.9	3.2	4.1	5.2	6.1	6.8
Ride cost per 1000 ($40/ride)	[Reference]	786	2321	3758	5128	6434	7662	8816
Ride cost per 1000 ($100/ride)	[Reference]	1965	5802	9395	12 820	16 086	19 155	22 039
Maximum cost per ride	[Reference]	92	219	286	317	344	369	361
Net costs per 1000 ($40/ride)	[Reference]	−6076	−22 402	−38 846	−52 956	−67 762	−83 277	−90 514
Net costs per 1000 ($100/ride)	[Reference]	−4897	−18 921	−33 208	−45 264	−58 111	−71 784	−77 290

Assuming a screening population of patients aged 55 years, a $40 rideshare intervention that increased colonoscopy completion by 15 pp (35% to 50%) was associated with a reduction in CRC cases per 1000 by 12.4% (31.7 vs 36.2 cases per 1000) and CRC deaths per 1000 by 17.1% (10.7 vs 12.9 cases per 1000), resulting in 8.5 LYG per 1000. An intervention that doubled colonoscopy completion in patients aged 55 years (from 35% to 70%) was associated with a reduction in CRC cases per 1000 by 24.3% (27.4 vs 36.2 cases per 1000), CRC deaths per 1000 by 31.8% (8.8 vs 12.9 cases per 1000) and resulted in 16.5 LYG per 1000 (Figure 1, Table). Model-estimated values with 95% CrIs are summarized in eTable 3 in Supplement 1.

The number of CRC cases and deaths averted was similar for the cohorts aged 45 and 55 years (less than 2% difference in the number of cancer cases and deaths averted), reflecting the relatively low CRC incidence before age 55 years. In comparison, there was a bigger difference in benefits between the cohorts aged 65 years and 70 years (maximum difference of 5.6% and 6.2% cases and deaths averted respectively for the cohorts aged 65 years vs 70 years assuming 70% adherence to follow-up colonoscopy completion). These differences are associated with increasing CRC incidence with age.

**Figure 2.**
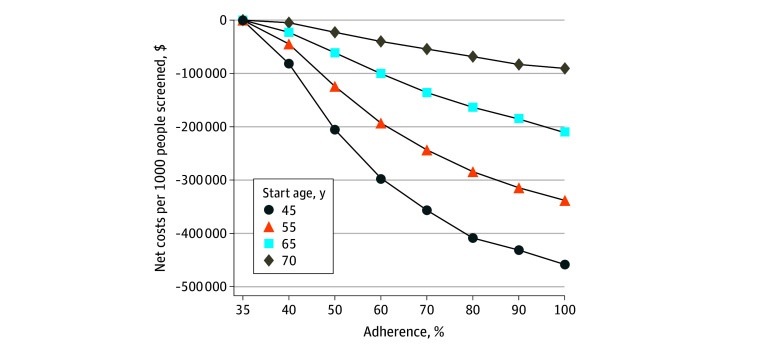
Net Lifetime Costs for Colorectal Cancer Screening at a Mean Cost of $40 Per Ride

**Figure 3.**
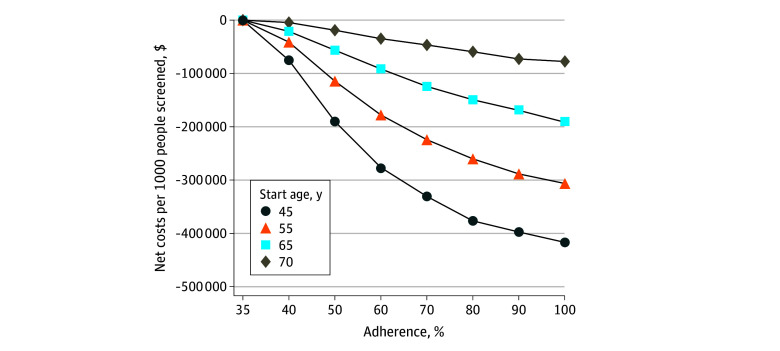
Net Lifetime Costs for Colorectal Cancer Screening at a Mean Cost of $100 Per Ride

While the magnitude of benefit decreased with age, intervention costs also decreased with age as there were fewer future colonoscopies. The intervention was cost-effective and cost-saving for all but the cohort aged 75 years (Figures 2 and 3), with 95% CrIs for net costs that fell below 0 (eTable 3 in Supplement 1). At $40 per ride, an intervention starting at age 45 years that increased colonoscopy completion by 15 pp (35% to 50%) was associated with saving $204 640 per 1000 with a direct lifetime cost of $8564 per 1000; when colonoscopy completion doubled (35% to 70%), the intervention was associated with saving $356 572 and had a direct lifetime cost of $17 323 (Table, Figure 2). At $100 per ride, an intervention starting at age 45 years that increased colonoscopy completion by 15 pp (35% to 50%) was associated with saving $191 794 per 1000 with a direct lifetime cost of $21 410 per 1000; when colonoscopy completion was doubled (35% to 70%), the intervention was associated with saving $330 587 and had a direct lifetime cost of $43 308 (Table, Figure 3; eTable 3 in Supplement 1).

In the cohort aged 45 years, when colonoscopy completion increased by 15 percentage points (35% to 50%) the intervention would remain cost saving if the mean ride cost was less than $316. In this same population, if colonoscopy completion doubled (35% to 70%), the intervention would remain cost saving if the mean ride cost was less than $418. In a cohort of 70-year-olds, if colonoscopy completion increased by 15 percentage points (35% to 50%), the intervention would remain cost saving if the mean ride cost was less than $219 and if colonoscopy completion doubled, the intervention would remain cost saving if the mean ride cost was less than $317 (Table). The rideshare intervention could cost up to a maximum of $498 per ride and remain cost saving (Table).

## Discussion

Using a microsimulation model, we demonstrated that increasing colonoscopy completion in a population with abnormal FIT results via a rideshare intervention was associated with reduced CRC incidence and mortality and would be cost saving at a mean cost of $40 or $100 per ride. Our results suggest that depending on the age at which screening begins and the magnitude of follow-up colonoscopy completion, the rideshare intervention could cost up to a maximum of $498 per ride and remain cost saving. Noninvasive CRC screening in the US is cost saving because it prevents CRC, avoiding higher CRC treatment costs ($2.5 billion vs $21.35 billion).^17,18^ Therefore, improving follow-up colonoscopy after abnormal screening tests is essential for reducing CRC incidence and death and reducing the costs of cancer care.

Ensuring follow-up of abnormal noninvasive cancer screening tests is also increasingly important given advances in single cancer and multicancer blood-based detection assays. Blood-based assays might potentially increase overall screening participation which has been a persistent challenge across many screening programs, including CRC.^19,20^ Yet, 1 study found that a FIT screening program with lower initial adherence (45%) but high follow-up colonoscopy completion (80%), had greater benefit at a lower cost than a blood-based test with perfect adherence.^21^ These findings highlight that without effective follow-up of abnormal results, the potential advantages of increased CRC screening through emerging blood-based tests may not be fully realized.

Notably, there are multiple barriers to colonoscopy after abnormal stool-based tests, including lack of transportation and/or chaperones; yet interventions to address this challenge are limited, especially in populations that experience the greatest disease burden.^22^ In the US, Black and American Indian or Alaskan Native populations have the highest CRC-mortality, yet have lower follow-up colonoscopy completion rates.^4,23^ One modeling study concluded that if Black adults had the same follow-up colonoscopy rates as White adults, CRC incidence and mortality would decrease by 5.2% and 9.3%, respectively, resulting in a 3.4% improvement in LYG (256 to 265).^24^ Yet, most studies aiming to improve follow-up colonoscopy to date have only focused on patient navigation, which increased follow-up colonoscopy completion by a mean (range) 13.6 (5.0-21.1) percentage points.^22,25^ Implementation details to support widespread adoption of navigation programs that improve follow-up are also direly needed.

While transportation is a benefit for some commercially insured and Medicare Advantage enrollees, these programs often face challenges due to limited access, geographic coverage, and scheduling complexity.^26^ A rideshare intervention is a potentially promising alternative due to widespread availability and ability to integrate into electronic health record platforms. Health care systems could use rideshare to complement existing transportation benefits by deploying this intervention for specific patient populations based on access or medical indication.

A 2023 federal policy eliminated cost-sharing for patients who required a colonoscopy after an abnormal noninvasive CRC screening test. A modeling study that assessed Medicare coverage of this policy estimated that a 15% increase in follow-up colonoscopy for all patients would improve LYG by 6%.^27^ Our study found that a modest 15% improvement in follow-up colonoscopy with a rideshare intervention was associated with 13.4 LYG per 1000 among a 45-year-old cohort and the effectiveness and cost-effectiveness of the intervention increased with greater adherence to follow-up colonoscopy completion. While eliminating procedural cost-sharing was an important first step, we propose that interventions that help patients overcome logistical barriers to follow-up colonoscopy should also be considered in future Medicare coverage decisions.

### Limitations

There are limitations to this study. First, as with any model-based analysis, the accuracy of our estimations is dependent on the validity of the baseline assumptions and estimates used (eTables 1 and 2 in Supplement 1). We believe our model assumptions are sound based on the validity of the model, preliminary data from the rideshare interventions, published literature, and multiple scenarios modeled.^7,8,13^ Second, this cost-effectiveness analysis precedes a large randomized clinical efficacy trial of the rideshare intervention. To address this limitation, we modeled multiple scenarios across multiple age groups to reflect currently available data. The $40 per ride is on par with published rideshare costs for other health indications (range, $14.00-$67.00).^7^ Our model also assumed a higher cost per ride ($100) to account for remote locations and thus provides a conservative estimate of potential cost-savings. Indeed, modeling studies can provide valuable insights to inform the design and conduct of larger efficacy trials. Third, our model assumed a baseline follow-up colonoscopy completion rate of 35% based on our health system’s prior experience. The effects and costs projected would differ for health systems with lower or higher baseline follow-up colonoscopy completion rates. However, up to a 37% improvement in colonoscopy completion is plausible based on rideshare studies in other medical settings.^8^ Fourth, our model assumes perfect adherence to surveillance and that with increased follow-up, colonoscopy services would be available to anyone who wanted to complete this procedure after an abnormal FIT. Model projections assumed that the rideshare costs would apply to both the follow-up and surveillance colonoscopies, but benefits could be lower than projected if adherence to surveillance was imperfect. Fifth, our model does not take into consideration programmatic or beneficiary costs that might accrue from the implementation of the rideshare intervention. In our health system, and likely others, identifying patients with transportation barriers is embedded in the clinical workflow at the point of colonoscopy scheduling.^6^ Additionally, requesting the rideshare replaces an existing phone call made by the endoscopy discharge team. For these reasons, programmatic costs are likely low, and in similar settings, a rideshare intervention would be cost saving or at least resource saving. Ultimately, we assumed that insurers would bear rideshare costs, but it is possible that some costs could be attributed to the beneficiary.

## Conclusions

In a simulation model study, we found that rideshare may be an effective and cost-effective intervention to improve follow-up colonoscopy after abnormal noninvasive CRC screening tests. Clinical trials are needed to validate the association between a rideshare intervention and follow-up colonoscopy completion. The results of this study will be used to identify target populations and to define study outcome goals in a forthcoming randomized clinical trial that includes a rideshare intervention. Future examinations of this intervention could also include assessments of how programmatic costs (eg, health systems that use intensive navigation) and use with novel blood-based tests might influence the intervention’s effectiveness and cost-effectiveness.^20^ With limited existing interventions to address this ever-growing challenge in cancer control, our results offer a potentially practical, scalable, and widely available intervention that could ultimately improve colorectal cancer care quality.

## Notes

Supplement 1. **eFigure.** Diagram of the CRCSPIN Microsimulation Model **eTable 1.** Parameters Used in the Model and References **eTable 2. **Costs and Disutility for FIT Tests, Colonoscopies, CRC Care, Colonoscopy Complications and Rideshare **eTable 3.** Colorectal Cancer Cases and Deaths Prevented, Life-Years Gained, Net Costs, and Maximum Cost per Ride by Varying Colonoscopy Adherence Across Multiple Age Groups With 95% CI
eReferences.

Supplement 2.
Data Sharing Statement
