# Supplementary material for: Modeled Cost-Effectiveness of a Rideshare Program to Facilitate Colonoscopy Completion
Source: JAMA Netw Open. 2025 Sep 4;8(9):e2530515. doi: 10.1001/jamanetworkopen.2025.30515 (PMC12411970; doi:10.1001/jamanetworkopen.2025.30515)
Supplement: Supplement 1. — eFigure. Diagram of the CRCSPIN Microsimulation Model eTable 1. Parameters Used in the Model and References eTable 2. Costs and Disutility for FIT Tests, Colonoscopies, CRC Care, Colonoscopy Complications and Rideshare eTable 3. Colorectal Cancer Cases and Deaths Prevented, Life-Years Gained, Net Costs, and Maximum Cost per Ride by Varying Colonoscopy Adherence Across Multiple Age Groups With 95% CI eReferences. [file jamanetwopen-e2530515-s001.pdf]

Supplemental Online Content

Issaka RB, Matrajt L, de Lima PN, Rutter CM. Modeled cost-effectiveness of a rideshare program to facilitate colonoscopy completion. *JAMA Netw Open*. 2025;8(9):e2530515. doi:10.1001/jamanetworkopen.2025.30515

eFigure. Diagram of the CRCSPIN Microsimulation Model
eTable 1. Parameters Used in the Model and References
eTable 2. Costs and Disutility for FIT Tests, Colonoscopies, CRC Care, Colonoscopy Complications and Rideshare
eTable 3. Colorectal Cancer Cases and Deaths Prevented, Life Years Gained, Net Costs, and Maximum Cost per Ride by Varying Colonoscopy Adherence Across Multiple Age Groups With 95% CI
eReferences.

This supplemental material has been provided by the authors to give readers additional information about their work.

© 2025 Issaka RB et al. *JAMA Network Open*.

eFigure. Diagram of the CRCSPIN Microsimulation Model

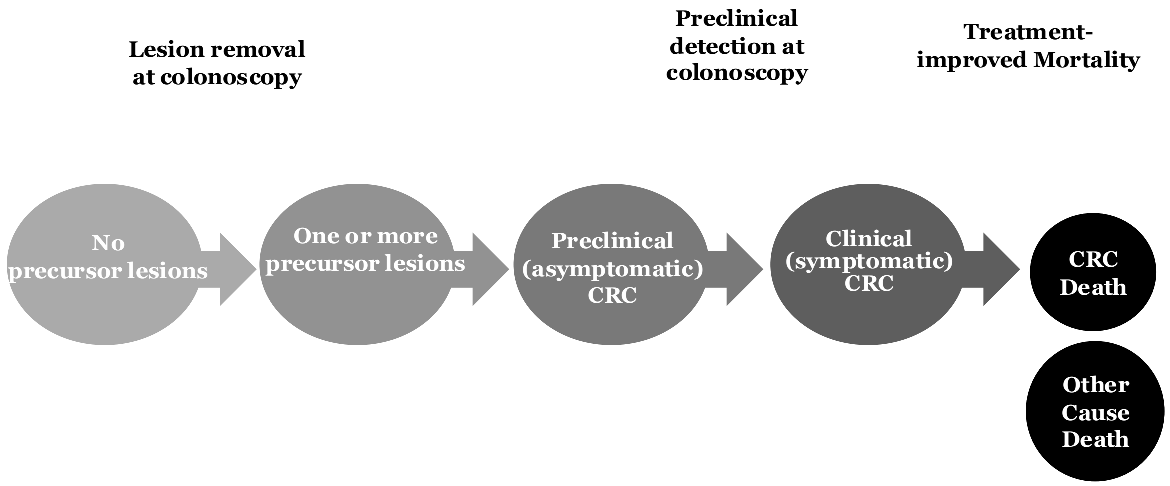

eFigure 1: Diagram of CRCSPIN, the microsimulation model used in this analysis. The model simulates 10 million individuals. For each individual, the natural history of CRC is simulated together with screening and treatment.

© 2025 Issaka RB et al. *JAMA Network Open*.

eTable 1. Parameters Used in the Model and References

Parameter	Value	Reference
Colonoscopy sensitivity		
Adenoma size between 1 and 5 mm	0.69	Zhao et al., Gastro, 2019[1]
Adenoma size between 6 and 9 mm	0.81	Zhao et al., Gastro, 2019[1]
Adenoma size over 10 mm	0.91	Zhao et al., Gastro, 2019[1]
CRC	0.91	Zhao et al., Gastro, 2019[1]
Colonoscopy Specificity	0.86	Zhao et al., Gastro, 2019[1]
FIT sensitivity		
Adenoma size between 1 and 5 mm	0.05	Imperiale et al, NEJM 2014[2]
Adenoma size between 6 and 9 mm	0.15	Imperiale et al, NEJM 2014[2]
Adenoma size over 10 mm	0.22	Imperiale et al, NEJM 2014[2]
CRC	0.74	Imperiale et al, NEJM 2014[2]
FIT Specificity	0.97	Imperiale et al, NEJM 2014[2]

© 2025 Issaka RB et al. *JAMA Network Open*.

eTable 2. Costs and Disutility for FIT Tests, Colonoscopies, CRC Care, Colonoscopy Complications and Rideshare

A. Screening tests

**Test**	*Assumed costs for people under 65 yo*	*Assumed costs for people 65 yo and over*	*Disutility when positive*	*Disutility when negative*
**Follow-up or surveillance Colonoscopy**[a]				
Without lesion removal[b]	1,427.67	963.95	NA	0.000496
With lesion removal	1,889.78	1312.36	0.001401	NA
**FIT**[c]	25.53	23.42	0.00133	0.000063

B. Colorectal cancer care

**2007-2013 Commercial* costs per LY CRC care**[d]	*Initial care*	*Continuing care*	*Terminal care Death CRC*	*Terminal care Death OC*
Stage I CRC	55,570	78,801	114,491	170,133
Stage II CRC	5,719	6,650	10,289	48,776
Stage III CRC	112,144	126,413	132,346	166,423
Stage IV CRC	29,452	31,693	43,327	104,221
**2007-2013 CMS costs per LY CRC care**[d]				
Stage I CRC	41,163	58,372	84,809	126,025
Stage II CRC	4,236	4,926	7,622	36,130
Stage III CRC	83,069	93,639	98,034	123,308
Stage IV CRC	21,816	23,476	32,094	77,200
**Utility loss per LY with CRC care**[d,e]				
Stage I CRC	0.12	0.05	0.7	0.05
Stage II CRC	0.18	0.05	0.7	0.05
Stage III CRC	0.24	0.24	0.7	0.24
Stage IV CRC	0.7	0.7	0.7	0.7

C. Colonoscopy complications

Event	*Assumed costs for people under 65 yo*	*Assumed costs for people 65 yo and over*	*Utility loss*
Serious gastrointestinal event[f]	11,715.00	8,677.80	0.0055
Other gastrointestinal event[g]	8,862.00	6,564.40	0.0027
Cardiovascular event[h]	9,541.60	7,0678	0.0048

D. Rideshare costs

	Cost	Reference
Rideshare	$40 or $100	Assumed

Table adapted from van den Puttelaar et al, Gastro, 2024[3]; OC – other cause

*We used commercial costs for individuals younger than 65, and Centers for Medicare and Medicaid Services (CMS) costs for individuals older than 65.[4] As no data are available to inform commercial cost estimates for colonoscopy complications and CRC treatment, CMS rates were multiplied by 1.35 for individuals younger than age 65 years based on the observed mean ratio of commercial to Medicare payment rates for colorectal tests.[5]

a. Colonoscopy costs were based on an analysis of 2014 CMS data and inflated to 2021 USD using the 2021 Personal Health Care Deflator Price Index.

b. Used for colonoscopies performed as a diagnostic follow-up after a positive non-colonoscopy test and for surveillance colonoscopies.

c. FIT costs were based on 2021 Clinical Laboratory Fee Schedule data and inflated to 2021 USD using the 2021 Personal Health Care Deflator Price Index.

© 2025 Issaka RB et al. *JAMA Network Open*.

d. Care for CRC was divided into three clinically relevant phases: initial, continuing, and terminal care. The initial care phase was defined as the first 12 months after diagnosis; the terminal care phase was defined as the final 12 months of life; the continuing care phase was defined as all months in between. In the terminal care phase, we distinguished between CRC patients dying from CRC and CRC patients dying from another cause. For patients surviving less than 24 months, the final 12 months were allocated to the terminal care phase, and the remaining months were allocated to the initial care phase.

e. Utility losses for life years (LYs) with initial care were derived from a study by Ness et al.[6] For LYs with continuing care for stage I and II CRC, we assumed a utility loss of 0.05 QALYs; for LYs with continuing care for stage III and IV CRC, we assumed the corresponding utility losses for LYs with initial care. For LYs with terminal care for CRC, we assumed the utility loss for LYs with initial care for stage IV CRC. For LYs with terminal care for another cause, we assumed the corresponding utility losses for LYs with continuing care.

f. Serious gastrointestinal events are perforations, gastrointestinal bleeding, or transfusions. The rate depends on age, formula: 1/[exp(9.27953 - 0.06105 x Age) + 1] - 1/[exp(10.78719 - 0.06105 x Age) + 1].

g. Other gastrointestinal events are paralytic ileus, nausea and vomiting, dehydration, or abdominal pain. The rate depends on age, formula: 1/[exp(8.81404 - 0.05903 x Age) + 1] - 1/[exp(9.61197 - 0.05903 x Age) + 1].

h. Cardiovascular events are myocardial infarction or angina, arrhythmias, congestive heart failure, cardiac or respiratory arrest, syncope, hypotension, or shock. The rate depends on age, formula: 1/[exp(9.09053 - 0.07056 x Age) + 1] - 1/[exp(9.38297 - 0.07056 x Age) + 1].

© 2025 Issaka RB et al. *JAMA Network Open*.

eTable 3. Colorectal Cancer Cases and Deaths Prevented, Life Years Gained, Net Costs, and Maximum Cost per Ride by Varying Colonoscopy Adherence Across Multiple Age Groups With 95% CI

Starting Age	Outcome	Adherence 35	40	50	60	70	80	90	100
45	Cases per 1000	41.58	39.19	35.59	32.82	30.65	28.93	27.59	26.43
	95% CI	(35.82-50.33)	(33.26-47.96)	(29.71-43.94)	(27.27-40.57)	(25.37-37.80)	(23.84-35.73)	(22.59-34.01)	(21.67-32.81)
	Deaths per 1000	14.6	13.55	11.95	10.74	9.85	9.16	8.6	8.19
	95% CI	(12.61-17.94)	(11.44-16.69)	(9.87-14.78)	(8.85-13.30)	(8.06-12.22)	(7.48-11.41)	(7.03-10.75)	(6.66-10.24)
	Costs per 1000	$4,742,562.62	$4,658,502.87	$4,529,358.16	$4,432,232.28	$4,368,667.48	$4,313,548.22	$4,286,592.52	$4,258,049.34
	95% CI	(4234022.18-5563819.57)	(4177005.72-5480259.41)	(4069628.39-5319009.65)	(3992732.37-5200908.73)	(3939072.24-5091324.28)	(3900056.85-5016255.50)	(3872669.31-4951231.33)	(3866294.05-4924198.87)
	Ride Cost per 1000 ($40/ride)	$0.00	$3,106.71	$8,564.04	$13,230.35	$17,323.22	$20,960.00	$24,273.96	$27,294.17
	95% CI	(0.00-0.00)	(2880.52-3411.99)	(7985.10-9341.30)	(12406.46-14359.52)	(16314.11-18686.60)	(19829.48-22493.88)	(23036.28-25902.48)	(25972.00-28987.45)
	Ride Cost per 1000 ($100/ride)	$0.00	$7,766.79	$21,410.10	$33,075.87	$43,308.04	$52,400.00	$60,684.91	$68,235.42
	95% CI	(0.00-0.00)	(7201.30-8529.97)	(19962.76-23353.25)	(31016.14-35898.80)	(40785.28-46716.51)	(49573.70-56234.69)	(57590.70-64756.20)	(64930.00-72468.63)
	Net Cost per 1000 ($40/ride)	$0.00	-$80,953.03	-$204,640.41	-$297,099.98	-$356,571.92	-$408,054.40	-$431,696.14	-$457,219.11
	95% CI	(0.00-0.00)	(-116763.59 - -51425.44)	(-318016.32 - -137499.29)	(-467690.74 - -192822.33)	(-571226.49 - -234150.09)	(-654161.00 - -256673.24)	(-708442.12 - -272498.56)	(-749365.29 - -279657.12)
	Net Cost per 1000 ($100/ride)	$0.00	-$76,292.95	-$191,794.36	-$277,254.46	-$330,587.09	-$376,614.40	-$395,285.19	-$416,277.86
	95% CI	(0.00-0.00)	(-112028.43 - -46690.67)	(-304335.43 - -125373.09)	(-446949.54 - -173685.99)	(-544171.89 - -208621.57)	(-621505.12 - -225768.30)	(-671691.77 - -237119.11)	(-707098.81 - -239355.54)
	QALY Per 1000	$22,242.23	$22,248.61	$22,258.99	$22,267.22	$22,273.53	$22,278.71	$22,282.96	$22,286.26
	95% CI	(22218.01-22256.74)	(22225.16-22262.06)	(22237.38-22271.67)	(22246.53-22279.89)	(22255.55-22285.88)	(22261.44-22290.04)	(22267.10-22293.55)	(22271.82-22296.72)
	QALY Gained Per 1000	0	6.38	16.76	24.99	31.3	36.48	40.73	44.03
	95% CI	(0.00-0.00)	(5.26-8.20)	(14.13-21.89)	(21.14-32.24)	(26.58-40.56)	(30.99-46.84)	(34.54-52.05)	(37.53-56.16)
	Life Years Per 1000	22272.92	22278.02	22286.28	22292.77	22297.83	22301.78	22305.18	22307.59
	95% CI	(22254.98-22283.41)	(22261.11-22287.87)	(22270.95-22295.28)	(22278.19-22301.63)	(22284.87-22306.25)	(22289.32-22309.50)	(22293.79-22312.17)	(22297.33-22314.71)
	Life Years Gained Per 1000	0	5.11	13.36	19.85	24.92	28.87	32.27	34.67
	95% CI	(0.00-0.00)	(4.23-6.59)	(11.42-17.57)	(16.96-25.62)	(21.15-32.16)	(24.75-36.99)	(27.40-41.00)	(29.76-44.15)

© 2025 Issaka RB et al. *JAMA Network Open*.

eTable 3, continued

Starting Age	Outcome	Adherence 35	40	50	60	70	80	90	100
55	Cases per 1000	36.21	34.53	31.65	29.22	27.36	25.75	24.49	23.39
	95% CI	(32.15-41.30)	(30.52-39.27)	(27.67-35.86)	(25.52-33.18)	(23.74-31.10)	(22.23-29.48)	(21.02-28.22)	(19.94-27.07)
	Deaths per 1000	12.85	12.05	10.71	9.63	8.84	8.15	7.64	7.19
	95% CI	(11.27-14.87)	(10.50-13.90)	(9.20-12.34)	(8.19-11.14)	(7.43-10.23)	(6.85-9.53)	(6.37-8.91)	(5.98-8.40)
	Costs per 1000	$3,134,809.27	$3,087,200.39	$3,003,549.42	$2,931,317.64	$2,878,981.42	$2,835,524.10	$2,801,480.19	$2,777,638.16
	95% CI	(2868924.25-3476437.31)	(2830127.15-3406485.98)	(2751871.41-3288024.72)	(2689600.99-3200773.31)	(2638303.08-3139041.95)	(2602292.27-3088332.52)	(2575732.28-3050151.85)	(2555602.40-3021504.42)
	Ride Cost per 1000 ($40/ride)	$0.00	$2,258.75	$6,306.12	$9,839.42	$12,960.15	$15,765.68	$18,286.75	$20,620.44
	95% CI	(0.00-0.00)	(2133.08-2412.64)	(5954.76-6685.61)	(9319.03-10385.37)	(12334.50-13661.87)	(15045.60-16550.62)	(17493.72-19178.98)	(19738.37-21548.47)
	Ride Cost per 1000 ($100/ride)	$0.00	$5,646.88	$15,765.29	$24,598.55	$32,400.38	$39,414.21	$45,716.88	$51,551.09
	95% CI	(0.00-0.00)	(5332.71-6031.59)	(14886.91-16714.01)	(23297.58-25963.42)	(30836.25-34154.67)	(37614.00-41376.56)	(43734.30-47947.44)	(49345.93-53871.19)
	Net Cost per 1000 ($40/ride)	$0.00	-$45,350.12	-$124,953.73	-$193,652.20	-$242,867.69	-$283,519.48	-$315,042.32	-$336,550.67
	95% CI	(0.00-0.00)	(-63174.28 - -32598.14)	(-175021.39 - -96064.46)	(-261198.39 - -144316.54)	(-333736.86 - -181178.58)	(-396169.73 - -211925.15)	(-434022.12 - -232402.59)	(-473754.49 - -250067.94)
	Net Cost per 1000 ($100/ride)	$0.00	-$41,961.99	-$115,494.56	-$178,893.07	-$223,427.46	-$259,870.96	-$287,612.20	-$305,620.02
	95% CI	(0.00-0.00)	(-59772.24 - -29172.54)	(-165628.33 - -86761.23)	(-246795.54 - -129532.86)	(-314402.16 - -161701.67)	(-372644.26 - -188657.83)	(-406707.69 - -205412.44)	(-442940.22 - -219003.90)
	QALY Per 1000	13908.91	13912.85	13919.48	13925.21	13929.49	13933.14	13936.15	13938.86
	95% CI	(13897.95-13918.05)	(13902.67-13921.80)	(13910.38-13928.03)	(13916.57-13933.13)	(13922.04-13937.41)	(13925.99-13940.67)	(13929.38-13943.44)	(13932.43-13945.76)
	QALY Gained Per 1000	0	3.94	10.57	16.3	20.58	24.23	27.24	29.96
	95% CI	(0.00-0.00)	(3.37-4.77)	(9.47-12.69)	(14.27-18.96)	(18.31-24.42)	(21.51-28.66)	(24.34-32.37)	(26.59-35.25)
	Life Years Per 1000	13929.86	13933.08	13938.37	13942.94	13946.35	13949.18	13951.53	13953.7
	95% CI	(13921.71-13936.96)	(13925.39-13939.92)	(13931.76-13944.94)	(13936.66-13948.91)	(13941.00-13952.29)	(13944.22-13954.73)	(13946.76-13956.90)	(13949.03-13958.83)
	Life Years Gained Per 1000	0	3.23	8.51	13.08	16.49	19.32	21.67	23.84
	95% CI	(0.00-0.00)	(2.74-3.86)	(7.62-10.29)	(11.50-15.32)	(14.75-19.51)	(17.23-22.80)	(19.40-25.71)	(21.29-27.97)

© 2025 Issaka RB et al. *JAMA Network Open*.

eTable 3, continued

		Adherence							
Starting Age	Outcome	35	40	50	60	70	80	90	100
65	Cases per 1000<br>95% CI	30.75<br>(27.26-35.71)	29.74<br>(26.33-34.46)	27.9<br>(24.70-32.42)	26.33<br>(23.23-30.57)	24.95<br>(21.99-28.95)	23.78<br>(20.78-27.57)	22.68<br>(19.79-26.27)	21.74<br>(18.88-25.17)
	Deaths per 1000<br>95% CI	11.05<br>(9.72-13.00)	10.53<br>(9.19-12.36)	9.62<br>(8.44-11.35)	8.91<br>(7.75-10.45)	8.25<br>(7.16-9.73)	7.69<br>(6.66-9.06)	7.2<br>(6.21-8.54)	6.81<br>(5.88-8.04)
	Costs per 1000<br>95% CI	$1,768,754.57<br>(1595422.90-2008946.43)	$1,744,840.26<br>(1573202.43-1979229.37)	$1,702,892.31<br>(1538230.86-1920690.13)	$1,662,919.59<br>(1502816.32-1870895.09)	$1,625,142.99<br>(1472978.52-1823390.57)	$1,595,222.33<br>(1448106.90-1782933.42)	$1,571,168.81<br>(1424294.03-1742442.87)	$1,544,709.87<br>(1407316.13-1711883.24)
	Ride Cost per 1000 ($40/ride)<br>95% CI	$0.00<br>(0.00-0.00)	$1,337.84<br>(1244.86-1436.91)	$3,816.47<br>(3578.33-4109.58)	$6,084.86<br>(5684.77-6518.60)	$8,135.65<br>(7621.72-8680.97)	$10,010.33<br>(9416.25-10674.71)	$11,740.62<br>(11066.97-12503.14)	$13,356.16<br>(12616.73-14180.27)
	Ride Cost per 1000 ($100/ride)<br>95% CI	$0.00<br>(0.00-0.00)	$3,344.60<br>(3112.14-3592.28)	$9,541.18<br>(8945.82-10273.96)	$15,212.14<br>(14211.94-16296.50)	$20,339.12<br>(19054.29-21702.43)	$25,025.82<br>(23540.61-26686.78)	$29,351.55<br>(27667.42-31257.85)	$33,390.39<br>(31541.82-35450.67)
	Net Cost per 1000 ($40/ride)<br>95% CI	$0.00<br>(0.00-0.00)	-$22,576.46<br>(-30683.02 - -14868.19)	-$62,045.79<br>(-84787.41 - -46483.71)	-$99,750.12<br>(-132023.97 - -76103.84)	-$135,475.94<br>(-176784.81 - -102024.77)	-$163,521.91<br>(-213463.31 - -124648.18)	-$185,845.14<br>(-252450.12 - -145381.49)	-$210,688.54<br>(-278831.51 - -161647.61)
	Net Cost per 1000 ($100/ride)<br>95% CI	$0.00<br>(0.00-0.00)	-$20,569.70<br>(-28743.62 - -12742.05)	-$56,321.09<br>(-78755.36 - -40975.19)	-$90,622.83<br>(-122982.92 - -66622.41)	-$123,272.47<br>(-164911.69 - -89892.35)	-$148,506.43<br>(-198836.59 - -109716.43)	-$168,234.21<br>(-235262.38 - -127090.24)	-$190,654.31<br>(-259298.81 - -142061.67)
	QALY Per 1000<br>95% CI	8157.5<br>(8149.07-8162.85)	8159.31<br>(8151.39-8164.54)	8162.33<br>(8154.98-8167.08)	8165.06<br>(8158.09-8169.66)	8167.58<br>(8160.88-8172.02)	8169.71<br>(8163.17-8174.04)	8171.47<br>(8165.16-8175.61)	8173.08<br>(8166.89-8176.88)
	QALY Gained Per 1000<br>95% CI	0<br>(0.00-0.00)	1.82<br>(1.48-2.14)	4.83<br>(4.22-5.85)	7.56<br>(6.65-9.01)	10.08<br>(8.75-11.89)	12.21<br>(10.65-14.29)	13.98<br>(12.24-16.48)	15.59<br>(13.58-18.36)
	Life Years Per 1000<br>95% CI	8170.08<br>(8163.62-8174.12)	8171.59<br>(8165.36-8175.54)	8174.05<br>(8168.16-8177.72)	8176.24<br>(8170.71-8179.86)	8178.23<br>(8172.87-8181.51)	8179.96<br>(8174.57-8183.09)	8181.39<br>(8176.13-8184.36)	8182.59<br>(8177.46-8185.34)
	Life Years Gained Per 1000<br>95% CI	0<br>(0.00-0.00)	1.51<br>(1.21-1.75)	3.97<br>(3.44-4.77)	6.16<br>(5.42-7.37)	8.16<br>(7.11-9.67)	9.88<br>(8.60-11.57)	11.31<br>(9.86-13.31)	12.52<br>(10.91-14.82)

© 2025 Issaka RB et al. *JAMA Network Open*.

eTable 3, continued

Starting Age	Outcome	Adherence 35	40	50	60	70	80	90	100
70	Cases per 1000<br>95% CI	26.59<br>(23.70-31.31)	26.08<br>(23.14-30.66)	25.01<br>(22.08-29.36)	24.03<br>(21.25-28.22)	23.14<br>(20.40-27.23)	22.29<br>(19.59-26.23)	21.48<br>(18.81-25.26)	20.82<br>(18.16-24.41)
	Deaths per 1000<br>95% CI	9.79<br>(8.69-11.61)	9.5<br>(8.44-11.29)	8.97<br>(7.88-10.59)	8.44<br>(7.43-10.07)	8.05<br>(7.00-9.53)	7.62<br>(6.64-9.06)	7.26<br>(6.32-8.59)	6.96<br>(6.00-8.23)
	Costs per 1000<br>95% CI	$1,245,440.84<br>(1125585.95-1441642.95)	$1,238,578.88<br>(1117576.04-1428503.14)	$1,220,717.97<br>(1101742.20-1398601.94)	$1,202,837.06<br>(1083601.19-1376520.20)	$1,187,356.47<br>(1070412.67-1354655.86)	$1,171,244.03<br>(1056131.84-1332701.38)	$1,154,501.53<br>(1041329.08-1309295.16)	$1,146,111.49<br>(1029355.26-1290568.79)
	Ride Cost per 1000 ($40/ride)<br>95% CI	$0.00<br>(0.00-0.00)	$785.94<br>(740.86-864.41)	$2,320.79<br>(2169.68-2532.02)	$3,758.17<br>(3514.58-4065.59)	$5,128.18<br>(4789.31-5540.52)	$6,434.36<br>(6005.97-6901.58)	$7,662.01<br>(7163.20-8224.08)	$8,815.72<br>(8263.21-9435.94)
	Ride Cost per 1000 ($100/ride)<br>95% CI	$0.00<br>(0.00-0.00)	$1,964.84<br>(1852.14-2161.02)	$5,801.97<br>(5424.20-6330.05)	$9,395.42<br>(8786.45-10163.98)	$12,820.44<br>(11973.28-13851.31)	$16,085.91<br>(15014.92-17253.95)	$19,155.03<br>(17908.00-20560.20)	$22,039.29<br>(20658.02-23589.86)
	Net Cost per 1000 ($40/ride)<br>95% CI	$0.00<br>(0.00-0.00)	-$6,076.02<br>(-13455.57 - -2453.90)	-$22,402.08<br>(-35776.46 - -15286.87)	-$38,845.61<br>(-58139.75 - -26170.82)	-$52,956.19<br>(-82013.50 - -40199.67)	-$67,762.45<br>(-102673.10 - -49290.78)	-$83,277.30<br>(-124910.96 - -60677.81)	-$90,513.63<br>(-142510.14 - -71488.21)
	Net Cost per 1000 ($100/ride)<br>95% CI	$0.00<br>(0.00-0.00)	-$4,897.12<br>(-12310.45 - -1154.48)	-$18,920.90<br>(-32244.94 - -11681.35)	-$33,208.36<br>(-52652.06 - -20667.93)	-$45,263.93<br>(-74553.10 - -32675.69)	-$58,110.90<br>(-93335.97 - -39503.39)	-$71,784.28<br>(-113755.72 - -49209.68)	-$77,290.05<br>(-129664.11 - -57714.51)
	QALY Per 1000<br>95% CI	6001.5<br>(5995.69-6005.70)	6002.28<br>(5996.66-6006.47)	6003.82<br>(5998.37-6008.01)	6005.34<br>(6000.09-6009.47)	6006.51<br>(6001.45-6010.74)	6007.81<br>(6002.81-6011.89)	6008.92<br>(6004.05-6012.85)	6009.83<br>(6005.18-6013.85)
	QALY Gained Per 1000<br>95% CI	0<br>(0.00-0.00)	0.78<br>(0.62-1.02)	2.32<br>(2.09-2.85)	3.85<br>(3.37-4.53)	5.02<br>(4.57-6.11)	6.31<br>(5.65-7.58)	7.42<br>(6.63-8.89)	8.34<br>(7.54-10.10)
	Life Years Per 1000<br>95% CI	6010.13<br>(6005.47-6013.80)	6010.79<br>(6006.31-6014.45)	6012.06<br>(6007.64-6015.75)	6013.33<br>(6009.02-6016.81)	6014.27<br>(6010.19-6017.91)	6015.33<br>(6011.20-6018.78)	6016.21<br>(6012.23-6019.61)	6016.98<br>(6013.20-6020.36)
	Life Years Gained Per 1000<br>95% CI	0<br>(0.00-0.00)	0.66<br>(0.54-0.85)	1.93<br>(1.75-2.40)	3.19<br>(2.80-3.78)	4.14<br>(3.80-5.01)	5.2<br>(4.66-6.24)	6.08<br>(5.48-7.24)	6.84<br>(6.18-8.21)

© 2025 Issaka RB et al. *JAMA Network Open*.

eTable 3, continued

Starting Age	Outcome	Adherence 35	40	50	60	70	80	90	100
75	Cases per 1000	22.2 (19.64-25.79)	22.12 (19.56-25.67)	21.94 (19.38-25.46)	21.77 (19.23-25.27)	21.58 (19.09-25.05)	21.4 (18.91-24.84)	21.23 (18.74-24.64)	21.08 (18.59-24.42)
	Deaths per 1000	8.42 (7.41-9.78)	8.36 (7.36-9.70)	8.26 (7.26-9.57)	8.15 (7.16-9.45)	8.03 (7.07-9.32)	7.92 (6.96-9.20)	7.81 (6.86-9.07)	7.71 (6.75-8.94)
	Costs per 1000	$795,108.58 (704401.92-921172.75)	$794,901.23 (704208.95-919332.04)	$795,441.35 (705075.07-919273.50)	$794,770.02 (705573.06-918108.07)	$793,624.98 (704508.49-916105.65)	$792,507.82 (704978.22-914015.85)	$792,182.48 (704446.00-911509.06)	$792,052.12 (704106.12-909476.24)
	Ride Cost per 1000 ($40/ride)	$0.00 (0.00-0.00)	$155.90 (144.12-167.00)	$472.39 (436.25-509.37)	$786.49 (725.91-851.93)	$1,098.38 (1015.15-1190.44)	$1,411.60 (1304.57-1527.98)	$1,723.52 (1596.74-1867.47)	$2,034.47 (1885.70-2204.33)
	Ride Cost per 1000 ($100/ride)	$0.00 (0.00-0.00)	$389.74 (360.29-417.50)	$1,180.97 (1090.62-1273.43)	$1,966.22 (1814.77-2129.82)	$2,745.94 (2537.86-2976.10)	$3,529.01 (3261.42-3819.96)	$4,308.79 (3991.85-4668.67)	$5,086.17 (4714.25-5510.83)
	Net Cost per 1000 ($40/ride)	$0.00 (0.00-0.00)	-$51.45 (-1189.53-705.16)	$805.16 (-3473.32-1494.38)	$447.93 (-4523.75-2094.67)	-$385.23 (-6603.45-2257.99)	-$1,189.16 (-7534.40-3032.25)	-$1,202.59 (-9689.45-3219.50)	-$1,021.99 (-11487.62-3937.66)
	Net Cost per 1000 ($100/ride)	$0.00 (0.00-0.00)	$182.39 (-971.71-939.89)	$1,513.74 (-2805.82-2238.05)	$1,627.66 (-3387.81-3252.48)	$1,262.34 (-4950.54-4043.65)	$928.25 (-5465.88-5373.29)	$1,382.69 (-7149.88-6098.17)	$2,029.71 (-8532.36-7032.51)
	QALY Per 1000	4244.57 (4240.53-4247.80)	4244.7 (4240.70-4247.92)	4244.92 (4240.99-4248.15)	4245.19 (4241.23-4248.40)	4245.45 (4241.47-4248.67)	4245.71 (4241.73-4248.93)	4245.95 (4242.00-4249.20)	4246.21 (4242.22-4249.49)
	QALY Gained Per 1000	0 (0.00-0.00)	0.12 (0.10-0.17)	0.34 (0.30-0.49)	0.62 (0.54-0.78)	0.88 (0.77-1.07)	1.13 (1.00-1.38)	1.38 (1.23-1.66)	1.64 (1.47-1.97)
	Life Years Per 1000	4250.02 (4246.48-4252.75)	4250.13 (4246.60-4252.88)	4250.34 (4246.83-4253.07)	4250.58 (4247.06-4253.29)	4250.82 (4247.28-4253.49)	4251.04 (4247.53-4253.73)	4251.25 (4247.76-4253.92)	4251.49 (4247.99-4254.16)
	Life Years Gained Per 1000	0 (0.00-0.00)	0.11 (0.09-0.15)	0.31 (0.27-0.44)	0.56 (0.48-0.69)	0.79 (0.69-0.96)	1.01 (0.91-1.22)	1.23 (1.12-1.48)	1.47 (1.33-1.72)

© 2025 Issaka RB et al. *JAMA Network Open*.

eReferences.
1. Zhao S, Wang S, Pan P, et al. Magnitude, Risk Factors, and Factors Associated With Adenoma Miss Rate of Tandem Colonoscopy: A Systematic Review and Meta-analysis. *Gastroenterology*. May 2019;156(6):1661-1674 e11. doi:10.1053/j.gastro.2019.01.260
2. Imperiale TF, Ransohoff DF, Itzkowitz SH. Multitarget stool DNA testing for colorectal-cancer screening. *N Engl J Med*. Jul 10 2014;371(2):187-8. doi:10.1056/NEJMc1405215
3. van den Puttelaar R, Nascimento de Lima P, Knudsen AB, et al. Effectiveness and Cost-Effectiveness of Colorectal Cancer Screening With a Blood Test That Meets the Centers for Medicare & Medicaid Services Coverage Decision. *Gastroenterology*. Jul 2024;167(2):368-377. doi:10.1053/j.gastro.2024.02.012
4. Nascimento de Lima P, van den Puttelaar R, Knudsen AB, et al. Characteristics of a cost-effective blood test for colorectal cancer screening. *J Natl Cancer Inst*. Oct 1 2024;116(10):1612-1620. doi:10.1093/jnci/djae124
5. Ladabaum U, Mannalithara A, Brill JV, Levin Z, Bundorf KM. Contrasting Effectiveness and Cost-Effectiveness of Colorectal Cancer Screening Under Commercial Insurance vs. Medicare. *Am J Gastroenterol*. Dec 2018;113(12):1836-1847. doi:10.1038/s41395-018-0106-8
6. Ness RM, Holmes AM, Klein R, Dittus R. Utility valuations for outcome states of colorectal cancer. *Am J Gastroenterol*. Jun 1999;94(6):1650-7. doi:10.1111/j.1572-0241.1999.01157.x

© 2025 Issaka RB et al. *JAMA Network Open*.
