# Supplementary material for: Modeled Cost-Effectiveness of a Rideshare Program to Facilitate Colonoscopy Completion
Source: JAMA Netw Open. 2025 Sep 4;8(9):e2530515. doi: 10.1001/jamanetworkopen.2025.30515 (PMC12411970; doi:10.1001/jamanetworkopen.2025.30515)
Supplement: Supplement 2. — Data Sharing Statement [file jamanetwopen-e2530515-s002.pdf]

Data Sharing Statement

Issaka. Modeled Cost-Effectiveness of a Rideshare Program to Facilitate Colonoscopy Completion. *JAMA Netw Open*. Published September 04, 2025. doi:10.1001/jamanetworkopen.2025.30515

Data
Data available: No

Additional Information
Explanation for why data not available: The model assumptions are proprietary to CISNET, however can be recreated if desired using publicly available data from published studies.
